# Commentary: Functional bowel complaints and the impact on quality of life after colorectal cancer surgery in the elderly

**DOI:** 10.3389/fonc.2023.1150181

**Published:** 2023-04-25

**Authors:** Cinara Sacomori, Mónica Belén Martinez-Mardones, Luz Alejandra Lorca, Laura Isabel Solé

**Affiliations:** ^1^Facultad de Medicina Clínica Alemana, Universidad del Desarrollo, Santiago, Chile; ^2^Servicio de Salud Metropolitano Oriente, Hospital del Salvador, Santiago, Chile; ^3^Facultad de Medicina, Universidad Finis Terrae, Santiago, Chile; ^4^Consultorio de Motilidad Digestiva (CMD), Buenos Aires, Argentina

**Keywords:** low anterior resection syndrome (LARS), colorectal cancer, questionnaire, anorectal dysfunction, bowel symptoms

## 1 Introduction

We would like to congratulate Ketelaers et al. (2022) (1) on their article regarding prevalence of low anterior resection syndrome (LARS) complaints and the impact on quality of life in the elderly after colorectal cancer surgery. Their article concerns a population-based study of 1623 older adults who underwent colorectal cancer surgery with primary anastomosis. The study showed that, for rectal cancer patients, younger patients (<70 years) had a high probability to develop major LARS compared to older patients (≥70 years), with a prevalence of 57.3% and 40.6%, respectively, OR= 0.63 (p=0.04). For colon cancer patients, the study showed the prevalence of major LARS symptoms as similar between younger and older patients: 20.4% and 22.2%, respectively. In addition, patients with major LARS presented significantly impaired quality of life (1). This study used the LARS score questionnaire, which is a very popular tool among clinicians and researchers.

LARS is usually defined as a bowel function disorder after rectal resection impairing quality of life. A recently published consensus of experts and patients concluded that, to meet the definition of LARS, a patient must have had a sphincter-preserving rectal anterior resection and experience at least one of the following symptoms with one consequence. Symptoms included: variable/unpredictable bowel function, altered stool consistency, increased stool frequency, repeated painful stools, emptying difficulties, urgency, incontinence, and soiling. Possible consequences include: toilet dependence, preoccupation with bowel function, dissatisfaction with bowels, strategies and compromises, and impact on mental and emotional wellbeing, social and daily activities, relationships and intimacy, roles, commitments and responsibilities (2).

Recently, the term “LARS” has become more widely used, and studies have reported LARS-like symptoms not only among rectal cancer patients who underwent low anterior resection (LAR) (3, 4), but also among patients with sigmoid (5, 6), colon (7), and ovarian (8) cancers, as well as in non-cancer non-operative (5) patients. The Ketelaers et al. (2022) (1) study included patients with and without LAR. The previously mentioned studies have in common the use of the LARS score questionnaire and the detection of some degree of LARS among participants. The fact that the LARS score identifies people who have not undergone a LAR as presenting LARS suggests the questionnaire may lack specificity.

We do not agree with the use of the term “LARS” in reference to patients that have not been submitted to the specific surgical procedure, the LAR. We believe that doing so will create confusion among clinicians and researchers about what is being assessed or reported. Is it possible to refer to “LARS” (major, minor) when patients have not undergone a LAR? Thus, the aim of this commentary is to discuss the most appropriate terminology when reporting bowel/anorectal symptoms, when using the LARS score, among people who did not experience a LAR.

## 2 Discussion

The LARS score was proposed by Emmertsen and Laurberg (2012) (9), and submitted for international validation (10), with proposed normative data (11). A classification has been recommended for the total score: no LARS (0–20 points), minor LARS (21–29 points), and “major LARS” (30–42 points) (11). LARS score is a short and easy to apply/understand questionnaire. However, there is some discussion on its accuracy. Some studies showed a high prevalence of LARS among the general population, i.e., with preserved rectum, indicating low specificity but high sensitivity of the instrument (2).

It is worth noting that the items in the LARS score assess some aspects of bowel function that can feasibly be applied to many other health conditions, not only after a patient has undergone a LAR. Thus, there is a need for more studies on the validity of the LARS score in people with other health conditions. We agree with the use of the LARS score with other populations to assess bowel symptoms, but we do not agree with indicating that those people who have not undergone a LAR as presenting LARS (major, minor); because the latter is the diagnosis of a condition that is traditionally only associated with the LAR procedure.

There are other short questionnaires on the topic, such as the Wexner and Vaisey scales (**Table 1**). But these scales do not assess urgency, changes in the frequency of defecation, or the amount of accidental bowel leakage (soiling, moderate, or great amount of leakage), which are very common symptoms among colorectal cancer patients. The International Consultation on Incontinence Questionnaire Bowel Module (ICIQ-B) is a more complete questionnaire for the assessment of bowel function (12), including all the relevant aspects, but it is much longer than the previously mentioned scales.

**Table 1:** Comparison of items of LARS score, Wexner and Vaisey.

	LARS score	Wexner	Vaisey
Flatus continence	✓	✓	✓
Liquid Stool continence	✓	✓	✓
Solid stool continence		✓	✓
Frequency of defecation	✓		
Urgency to open bowels	✓		
Need to defecate 1 hour after last defecation	✓		
Wear a pad		✓	✓
Changes in lifestyle		✓	✓
Taking constipating medication			✓
Difficulty to defer defecation for 15 minutes			✓

For research purposes, we recommend the more comprehensive questionnaires (ICIQ-B, the Bowel Function Instrument of the Memorial Sloan Kettering Cancer Centre) that will assess more dimensions of the construct. For clinical purposes, the LARS score combined with other fecal incontinence scales (e.g., Wexner, Vaisey) and clinical assessment might be more feasible. However, we recommend interpreting only individual and general scores of the LARS score questionnaire to determine bowel/anorectal symptoms. The use of the classification of LARS (minor, major) should be restricted to patients who have undergone a LAR.

In addition, there is a need to standardize terminology to systematically assess and report symptoms, signs, and dysfunctions related to anorectal/bowel dysfunctions. In this effort, the International Continence Society (2019) proposed a classification of anorectal dysfunctions for males (13) and females (14) considering symptoms, signs, examination, and any relevant diagnostic investigations. In general, symptoms are classified as: anorectal incontinence (gas and fecal, liquid or solid), anorectal sensory symptoms (rectal hyposensitivity, rectal hypersensitivity), anorectal storage symptoms (increased frequency, urgency), defecatory or post-defecatory symptoms (constipation, feeling of incomplete evacuation, straining, manual defecatory assistance), anorectal pain, anorectal sexual dysfunction, miscellaneous, and anorectal prolapse (13, 14).

Meanwhile, the International Classification of Functioning, Disability and Health (ICF) terminology classifies these functions at category b5 (functions of the digestive, metabolic, and endocrine systems), subcategory b525 which is identified as defecation functions (functions of elimination, fecal consistency, frequency of defecation; fecal continence, flatulence; impairments such as constipation, diarrhea, watery stool and anal sphincter incompetence or incontinence).

In conclusion, a better definition or use of standardized terminology to describe anorectal/bowel function is imperative, mainly in topics that require interdisciplinary approaches (gastroenterologists, coloproctologists, surgeons, oncologists, nurses, and pelvic floor physiotherapists, among others). Interdisciplinary and patient-centered approaches as the consensus on the LARS definition, by Keane et al. (2020) (2), greatly contribute to the development of better care practices and comprehension of the LARS and other anorectal/bowel symptoms and dysfunctions.

## Author contributions

CS proposed the idea and wrote the manuscript. MM-M, LL and LS revised and contributed to the manuscript. All authors contributed to the article and approved the submitted version.
